# Strengthening mosquito control to manage emerging mosquito-borne diseases

**DOI:** 10.2807/1560-7917.ES.2026.31.33.2600306

**Published:** 2026-08-20

**Authors:** Olivier JT Briët, Antero Airaksinen, Laura Gillini, Maria Daniel Loureiro, Jeanne Vuaille, Vincent Delvaux, Ana Margarida Alho, Áine Collins, Céline M Gossner

**Affiliations:** 1 Food-, Water-, Vector-borne and Zoonotic Diseases Section, European Centre for Disease Prevention and Control (ECDC), Stockholm, Sweden; 2 Biocidal Active Substances, European Chemicals Agency (ECHA), Helsinki, Finland; 3 Unit B2 Health Security, Directorate-General for Health and Food Safety (DG SANTE), European Commission, Luxembourg, Luxembourg; 4 Unit 04 Threats & MCM Intelligence, Innovation and Funding, Health Emergency Preparedness and Response Authority (DG HERA), European Commission, Brussels, Belgium; 5 Freshwater and Marine, European Environment Agency (EEA), Copenhagen, Denmark; 6 Unit E4 Pesticides and Biocides, Directorate-General for Health and Food Safety (DG SANTE), European Commission, Brussels, Belgium

**Keywords:** Vector control, mosquito, dengue, chikungunya, West Nile virus infection, Biocidal Products Regulation, cost-effectiveness, governance

## Abstract

Mosquito-borne diseases such as dengue, chikungunya and West Nile virus infection become increasingly frequent in Europe. These developments require preparedness and coordinated response mechanisms across countries and sectors. Vector control is a central component of public health response, both for containing outbreaks and for reducing the risk of disease emergence. While mosquito control is important, evidence on the cost-effectiveness of most methods remains limited, complicating the design of evidence-based vector management strategies. In addition, the range of authorised vector control products is narrow and increasingly compromised by insecticide resistance. Mosquito control in European countries involves a diverse set of actors, and work on mosquito-borne diseases at European Union level spans several institutions with complementary mandates. Europe should therefore promote cross-sectoral collaboration through a One Health approach that brings together public health, environmental health, veterinary health and urban planning sectors, while engaging communities in mosquito surveillance and control. Continued investment in research to assess the cost-effectiveness, safety and ecological impact of existing and new vector control methods is needed to develop sustainable, science-based, integrated mosquito control strategies that protect public health, animal health and ecosystems. Regulatory clarity and flexibility, for biocidal, non-biocidal and innovative control approaches, would support this effort.

## Background

Mosquito-borne diseases are increasing globally, driven by expanding mosquito habitats, increased international travel, and changing environmental and climate conditions. While some viruses remain endemic to specific regions, others such as chikungunya and dengue viruses are increasingly emerging in new areas. Europe, where local transmission was previously rare, is now experiencing outbreaks.

In 2025, chikungunya virus disease, transmitted by the invasive [1] mosquito species *Aedes aegypti* and *Aedes albopictus*, surged: More than 445,000 suspected and confirmed cases were reported across 40 countries [2]; rapidly expanding outbreaks occurred in the Indian Ocean region and the Americas [2], while Europe has recorded an unprecedented number of locally acquired clusters, particularly in France and Italy, with 809 and 384 cases respectively [3]. Dengue virus, also transmitted by *Aedes* mosquitoes, is becoming more frequent in Europe. Reports of autochthonous cases have increased since the first records in 2010, reaching a peak in 2024, when 328 dengue cases were reported, the vast majority in Italy and France [4]. In Europe, dengue and chikungunya outbreaks typically start when infected travellers introduce viruses into areas with established (i.e. overwintering and reproducing) populations of invasive *Aedes* mosquitoes [5] and climatic conditions favourable for mosquito activity and virus replication in the mosquito. Dengue can result in severe complications such as haemorrhagic fever and shock syndrome, especially in individuals with prior infections. Chikungunya, although rarely fatal, can cause prolonged and debilitating joint pain that may persist for months or even years. Two chikungunya vaccines are available for populations with a high risk of infection, while only one dengue vaccine is currently approved for use in settings with high dengue transmission. However, protection from mosquito bites remains the primary measure for the prevention of both diseases. No specific antiviral treatment is available for dengue or chikungunya, limiting clinical management to symptomatic care.

West Nile virus (WNV), transmitted by native *Culex* mosquitoes, has been endemic in Europe since the 1960s, posing a persistent public health challenge. Unlike dengue and chikungunya viruses, WNV circulates locally via enzootic transmission between birds and mosquitoes; humans and equids can be infected but do not pass the virus back to mosquitoes and are therefore considered dead-end hosts. Severe neurological disease occurs in fewer than 1% of WNV infections [6]. The risk is highest among older and immunocompromised individuals, and case fatality among severe cases is around 10% [6]. The number of human cases varies from year to year. In 2018, the European Union (EU) recorded a peak of locally acquired WNV infections (1,549 cases) [4]. The virus continues to expand its geographical range, primarily through the movement of infected birds. In 2024, WNV reached its widest distribution to date, affecting 166 regions (NUTS-3 level) in 14 EU countries [7].

Europe also faces the emergence of other mosquito-borne pathogens, as changing environmental conditions and growing travel and trade create favourable conditions for importation and spread. Changes in land use and declining biodiversity influence the distribution of disease vectors and related disease risk, but the extent and nature of these effects vary depending on local ecological and environmental conditions. Sound land use and biodiversity management may help reduce disease risk [8], although such measures are not always feasible because they must be balanced against biodiversity conservation and other land-use priorities.

Strengthening preparedness and response capacity is essential, with mosquito control as a key pillar to limit transmission and reduce the impact of vector-borne outbreaks. Vector control is inherently complex, requiring multisectoral coordination across multiple actors. Yet, data to support safe, effective and cost-efficient implementation remain limited. This Perspective article highlights key challenges and gaps in vector control knowledge and strategies, while offering strategic avenues and perspectives to guide future actions. By promoting collaboration and a One Health approach, we aim to support the development of integrated and sustainable vector control strategies for Europe.

## Mosquito bite prevention

Mosquito bite prevention by individuals, via personal protective measures, is a core component of mosquito and mosquito-borne disease control. This can be done by applying topical and spatial repellents. Within the EU, the Biocidal Products Regulation (BPR, Regulation (EU) 528/2012) governs the placing on the market and use of biocidal products, including mosquito repellents and insecticides [9].

A biocidal product can be authorised if the active chemical substances or microorganisms they contain are approved for use, or under evaluation in an ongoing review programme examining all existing active substances contained in biocidal products [10]. However, under BPR Article 55(1), EU countries may temporarily allow unauthorised biocidal products on their markets under supervision. A search of the biocidal product database maintained by the European Chemicals Agency (ECHA) identified four active substances used in authorised mosquito repellent products under the BPR (the information of effectiveness against mosquitoes may not be comprehensive): IR3535, icaridin, margosa extract (only for application on (pet) animals indoors) and DEET [11].

Mosquito bites can also be prevented with non-biocidal methods such as sleeping under (untreated) bed nets, mosquito-proofing of homes with window and door screens, and wearing protective clothing. In the EU, building codes do not require insect screens as standard features on windows and doors, unlike in some states in the United States, such as California [12], so residents typically need to purchase and install them themselves.

## Mosquito population control

Mosquito populations can be reduced to lower nuisance levels and decrease the risk of vector-borne diseases by removing or treating larval habitats, such as stagnant water in containers, flooded basements, discarded tyres, catch basins or poorly drained land. When environmental management is not feasible, larvae can be controlled through biological methods (such as releasing natural predators, for example copepods and larvivorous fish), applying larvicides, or adding a physical film to the water surface. The active substances *Bacillus thuringiensis israelensis* (Bti), *Lysinibacillus sphaericus* (formerly *Bacillus sphaericus*), pyriproxyfen and methoprene are larvicides used in authorised products under the BPR to target aquatic stages.

During outbreaks, adult mosquitoes are often targeted through fogging or ultra-low-volume spraying with biocidal products to rapidly reduce vector populations. The search of the ECHA database identified 12 active substances in biocidal products authorised for use against adult mosquitoes, most of which are pyrethroids. Pyrethroids are synthetic chemicals modelled after the naturally occurring pyrethrins found in *Chrysanthemum* flowers. Among those 12, only deltamethrin, cyfluthrin, transfluthrin, cypermethrin and piperonyl butoxide (a synergist with limited insecticidal properties of its own that is used to enhance the effectiveness of pyrethroids) are contained in products authorised for outdoor use, while the others are restricted to indoor environments. Pyrethroids have a rapid paralysing effect (knockdown effect) on mosquitoes, but they degrade quickly in sunlight.

Techniques such as the sterile insect technique (SIT), *Wolbachia* symbionts and gene-drive technologies offer ways to reduce mosquito populations [13,14]. However, of these only SIT, which involves the release of mass-reared and sterilised males that mate with females to produce infertile eggs, is currently being piloted in Europe, at a limited scale, often in an integrated approach together with other vector control methods, for instance in France, Germany, Spain and Switzerland [15-18]. *Wolbachia* bacteria can reduce vector competence of infected females for *Aedes*-borne viruses. Native *Wolbachia* strains, meaning the *Wolbachia* bacteria that naturally occur in some populations of *Ae. albopictus*, such as those found in Réunion, have been shown to reduce dengue virus dissemination within the mosquito, limiting its ability to reach the salivary glands and be transmitted during feeding [19]. Similarly, *Ae. albopictus* experimentally infected with two *Wolbachia* strains showed a markedly reduced ability to transmit chikungunya and dengue viruses [20]. *Wolbachia* is under evaluation under the BPR, and its approval is a prerequisite for product authorisation. Gene-drive technologies are currently not permitted in the EU [21].

## Key challenges and gaps in vector control

There are many unknowns about the (relative) cost-effectiveness of these various methods to control mosquito populations and their associated diseases [22], making it difficult to design cost-effective mosquito control strategies. In addition, mosquito-borne diseases are not always perceived as a political priority, which can limit investment in surveillance and control, particularly in areas without local transmission or at the edge of vector invasion.

Environmental conditions for mosquito reproduction vary considerably across Europe, and control strategies that work in one setting may not be as effective in another. In addition, vector control methods rarely work in isolation and must be implemented with an integrated vector management approach, involving a combination of interventions tailored to local vector ecology, disease transmission patterns and available resources. The implementation of integrated vector management is further complicated by differences between countries regarding the availability of authorised biocidal products and in the conditions governing their use, which affect which vector control tools can be deployed in practice.

There is evidence that insecticide resistance in mosquitoes is increasing, and it is especially worrying that resistance against pyrethroids has been reported, as most biocidal products used for controlling adult mosquitoes rely on this class of chemicals [23]. This creates an urgent need to increase the number of vector control methods, including biocidal and non-biocidal approaches that are cost-effective, safe for human health and the environment, and socially acceptable. However, for biocidal products in particular, the relatively small European market and the high costs associated with obtaining authorisation under the BPR may delay new options.

## Environmental and health considerations

The widespread use of pyrethroids, mainly in agriculture, raises concerns about ecotoxicological and human health impacts. Cypermethrin is listed as a priority substance in the field of water policy following Directive 2013/39/EU [24], which amended the Water Framework Directive and the Environmental Quality Standards Directive. As a priority substance, cypermethrin is considered a significant risk to or via the aquatic environment, and hundreds of freshwater and marine water bodies across 11 EU countries fail to meet good chemical status because of its presence [25]. Pyrethroid exposure has also been linked to behavioural symptoms, neurodevelopmental disorders and prostate cancer [26,27], and biomonitoring data from the European Human Biomonitoring Initiative show widespread internal exposure across six European countries, with some biomarkers for pyrethroid exposure detected in more than 90% of samples [27]. However, the proportion of exposure attributable to mosquito control is unclear. A recent review summarised limited evidence on the effects pyrethroids used for mosquito control have on human health. In the few available studies, pyrethroids applied correctly for mosquito control were not linked to acute or chronic human health risks [28]. Further research is needed to better assess potential health risks related to mosquito control. Beyond pyrethroids, the larvicide Bti has been shown to disrupt wetland ecosystems by affecting the food web [29].

## Governance and implementation of mosquito control in European Union countries

Mosquito control in the EU countries is governed by a diverse set of actors. Surveys conducted by the European Centre for Disease Prevention and Control (ECDC) in 2020 and 2025 revealed that the Ministry of Health is most frequently the authority with overall responsibility for mosquito vector control [30,31]. In some countries, this responsibility is delegated to municipal authorities, while other ministries, such as those for environment, interior or agriculture, may also be involved. For example, ministries of agriculture often play a key role in controlling vectors of WNV.

However, operational control plans are typically designed at the sub-national level and executed by subcontracted private companies. Private individuals can be major contributors to vector control by applying personal protective measures and removing larval habitats in the peri-domestic environment.

These surveys also found that 20 of the 29 responding countries had implemented mosquito control measures [30,31]. All countries with local transmission of WNV and/or dengue virus reported undertaking control activities. Among the 12 countries with established mosquito vectors but no local transmission, four reported implementing preventive measures. Most countries focus on aquatic stages through biocidal or non-biocidal interventions, while a smaller number also target adult mosquitoes with biocides. Fewer countries use environmental management, social mobilisation programmes, non-biocidal adult control methods, or innovative approaches such as SIT. Control efforts are motivated by the need to prevent the establishment of invasive mosquitoes, reduce disease transmission risk and address mosquito nuisance.

## Governance and implementation of mosquito control at the European Union level

Work on mosquito-borne diseases at EU level involves several institutions with complementary mandates. Under Regulation (EU) 2022/2371 on serious cross-border threats to health, EU bodies and agencies collaborate to strengthen preparedness and response to cross-border health risks, including mosquito-borne diseases [32].

Several EU agencies contribute to assessment of and response to mosquito-borne diseases, according to their respective mandates. The ECDC and the European Food Safety Authority provide scientific assessments for public health and animal health, respectively. Their work includes providing scientific advice on mosquito surveillance and control as well as monitoring vector distribution through the joint VectorNet project. The ECHA evaluates active substances and biocidal products relevant for mosquito control, while the European Environment Agency provides environmental and climate data that contribute to assessing vector-borne disease risks.

Within the European Commission, the Directorate-General for Health and Food Safety oversees the implementation of EU health legislation and manages EU4Health actions that support vector surveillance and control, for example, a pilot trial assessing the operational use of SIT in response to the spread of *Ae. aegypti*. The Health Emergency Preparedness and Response Authority supports the development and availability of medical countermeasures; this includes an EU4Health action aiming to reinforce European countries’ capacity for vector detection and control. Research funding through the Horizon research and innovation framework programme further contributes to improving the evidence base for vector-borne disease assessment and response, and the Joint Research Centre develops analytical and modelling tools that support preparedness and response to mosquito-borne diseases.

Cooperation across these institutions is facilitated through multiple formal and informal mechanisms that enable information exchange, technical coordination, and alignment between EU agencies, Commission services and EU countries. Among these, the cross-agency One Health Task Force supports collaboration on threats that span environmental, animal and human health sectors [33].

Together, these activities contribute to a stronger and more coherent approach to addressing vector borne disease risks across Europe.

## Implications for mosquito control in the European Union

Mosquito control remains a critical component of outbreak prevention and mitigation. However, its implementation is challenged by fragmented governance, limited data on cost-effectiveness and safety of different measures, and a narrow range of approved biocidal active substances. Pyrethroids are effective biocides for adult mosquito control, but concerns remain about environmental and human health impacts from their wider use, as well as the risk of resistance.

To strengthen Europe’s capacity to manage mosquito populations, we recommend a number of measures (Box).

Box **Recommendations for managing mosquito populations**

- continued investment in research to evaluate the cost-effectiveness, safety and ecological impact of vector control methods, and to develop new ones;
- promoting integrated vector management strategies tailored to local ecological and epidemiological contexts;
- dedicated and sustained funding for the implementation of vector control measures, accompanied by regulatory clarity and flexibility, for biocidal, non-biocidal and innovative control approaches;
- cross-sectoral collaboration through a One Health approach involving public health, environmental, veterinary and urban planning sectors;
- engaging communities in mosquito surveillance and control efforts through education, mobilisation and support for peri-domestic interventions.

## Conclusion

Mosquito-borne diseases are an increasing public health challenge in Europe. Sustainable, evidence-based and coordinated mosquito control, implemented across subnational, national and EU levels and embedded in a One Health approach, will be essential to reduce outbreak risk while protecting public health, animal health and ecosystems.

## Data Availability

Data sharing is not applicable to this article as no datasets were generated or analysed during the current study.
